# Codon-Specific Translation by m^1^G37 Methylation of tRNA

**DOI:** 10.3389/fgene.2018.00713

**Published:** 2019-01-10

**Authors:** Ya-Ming Hou, Isao Masuda, Howard Gamper

**Affiliations:** Department of Biochemistry and Molecular Biology, Thomas Jefferson University, Philadelphia, PA, United States

**Keywords:** synonymous codons, methyl transferases TrmD and Trm5, codon-anticodon pairing interaction, ribosomal +1-frameshifts and stalling, protein synthesis

## Abstract

Although the genetic code is degenerate, synonymous codons for the same amino acid are not translated equally. Codon-specific translation is important for controlling gene expression and determining the proteome of a cell. At the molecular level, codon-specific translation is regulated by post-transcriptional epigenetic modifications of tRNA primarily at the wobble position 34 and at position 37 on the 3′-side of the anticodon. Modifications at these positions determine the quality of codon-anticodon pairing and the speed of translation on the ribosome. Different modifications operate in distinct mechanisms of codon-specific translation, generating a diversity of regulation that is previously unanticipated. Here we summarize recent work that demonstrates codon-specific translation mediated by the m^1^G37 methylation of tRNA at CCC and CCU codons for proline, an amino acid that has unique features in translation.

## Introduction

The redundancy of the genetic code offers an opportunity for fine-tuning of protein synthesis, virtually at every codon position. Although the sequence of codons in an mRNA provides the template for translation into amino acid building blocks of the protein, synonymous codons are not translated equally. Codon bias, which is defined by the frequencies of usage among synonymous codons, is a specific feature unique to each genome and each gene and can impact the fitness of each organism (Plotkin and Kudla, 2011). The choice of synonymous codons determines how these codons are differentially translated at the molecular level. Translation is controlled by tRNA species with the anticodon that is cognate to each codon. The quality of a codon-anticodon pairing interaction is determined not only by the level of the tRNA available for the codon (Ikemura, 1982; Komar, 2009; Nedialkova and Leidel, 2015), but also and perhaps more importantly by post-transcriptional modifications to the tRNA anticodon, either at nucleobases or backbones (Takai and Yokoyama, 2003; Armengod et al., 2014; Grosjean and Westhof, 2016; Agris et al., 2018). These modifications are catalyzed by distinct enzymes that bear high relevance to human health and disease (Sarin and Leidel, 2014). Many of these enzymes are dedicated to modifications of the anticodon at position 34 (the wobble position) and at position 37 on the 3′-side of the anticodon. It is the nature of post-transcriptional modifications at these anticodon-associated positions that exert differential pairing with synonymous codons, resulting in differential translation among these codons. The mechanism of codon-specific translation is therefore the underlying basis that determines how codon bias impacts the fitness of each organism. It is an emerging new concept that directly influences the expression of the proteome at the cell level.

## The m^1^G37-Methylation in tRna

**FIGURE 1:**
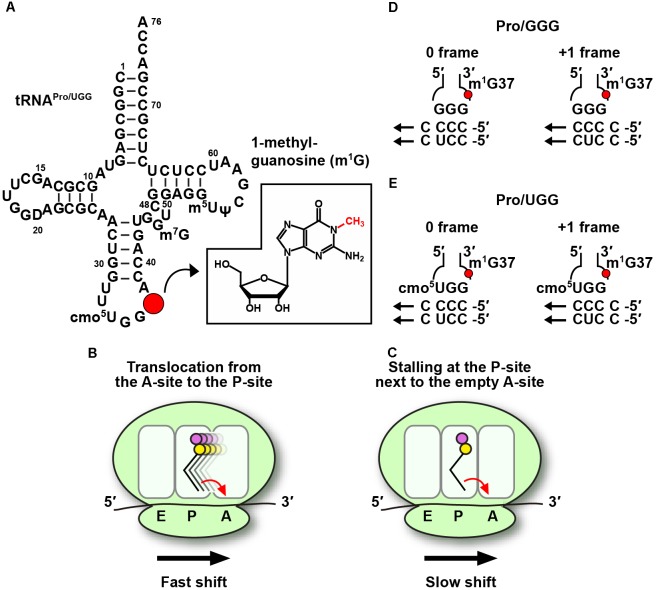
Codon-specific translation by m^1^G37-tRNA. **(A)** The sequence and cloverleaf structure of *Escherichia coli* UGG isoacceptor of tRNA^Pro^ (tRNA^Pro/UGG^) is shown with m^1^G37 indicated by a red circle and displayed in the chemical structure in the inset. **(B)** Lack of m^1^G37 promotes the tRNA to make +1-frameshifts in a fast mechanism during tRNA translocation from the A- to the P-site on the ribosome, and also **(C)** in a much slower mechanism during tRNA stalling on the P-site next to an empty A-site. The fast and slow mechanisms are marked in the figure for panels **B,C**, respectively. The arrow in each panel indicates the direction of the ribosome translocation in the 5′-to-3′ direction on the mRNA. The propensity of **(D)** the GGG isoacceptor and **(E)** the UGG isoacceptor of *E. coli* tRNA^Pro^ to make the +1-frameshift is based on similar base pairing stability with cognate codons CCC and CCU. The UGG isoacceptor contains the cmo^5^U34 modified base at the wobble position, which allows pairing with all four nucleobases.

While there are more than 100 post-transcriptional modifications in tRNA databases, the majority by themselves are not required for cell survival or growth. One exception is the *N*^1^-methylation of G37 on the 3′-side of the tRNA anticodon, generating m^1^G37 (Figure 1A), which as a single methylated nucleobase is not only essential for life but is also conserved in evolution present in all three domains of life (Bjork et al., 2001). In the bacterial domain, the biosynthesis of m^1^G37 is catalyzed by the tRNA methyl transferase TrmD (Hou et al., 2017), whereas in the eukaryotic and archaeal domains, it is catalyzed by Trm5 (Bjork et al., 2001). While both TrmD and Trm5 perform the same methyl transfer reaction, using *S*-adenosyl methionine (AdoMet) as the methyl donor, they are fundamentally different in structure, where TrmD is a member of the SpoU-TrmD family (Anantharaman et al., 2002; Ahn et al., 2003; Elkins et al., 2003; Ito et al., 2015; Hori, 2017) and Trm5 is a member of the Rossmann-fold family (Goto-Ito et al., 2008, 2009). TrmD and Trm5 also differ in virtually all aspects of the reaction mechanism (Christian et al., 2004, 2006, 2010a,b, 2013, 2016; Christian and Hou, 2007; Lahoud et al., 2011; Sakaguchi et al., 2012, 2014). Due to the dependence on m^1^G37 for cell survival, TrmD is required for growth in several bacterial species, including *Escherichia coli* and *Salmonella* (Gamper et al., 2015a), and Trm5 is required for growth in the single-cell eukaryote yeast *Saccharomyces cerevisiae* (Bjork et al., 2001), where it provides the important role of preventing mis-charging of tRNA (Perret et al., 1990). Additionally, the Trm5-dependent synthesis of m^1^G37 is present in both the cytosolic and mitochondrial compartments (Lee et al., 2007) and it is the initiation event that leads to further modifications to m^1^I37 (Brule et al., 2004) and to wyosine and derivatives in the cytosol (Urbonavicius et al., 2014). The molecular basis for how m^1^G37-tRNA is essential for cell survival is largely elucidated in *E. coli* and *Salmonella* (Gamper et al., 2015a).

## Maintenance of Protein Synthesis Reading Frame by m^1^G37-tRna

In bacteria, all three tRNA^Pro^ species, which read CCN codons, contain m^1^G37. Elimination of m^1^G37 by inactivation of TrmD leads to accumulation of ribosomal +1-frameshifts (+1-shifts), particularly at sites where mRNA sequences contain successive Cs in a row (Bjork et al., 1989). Unlike ribosomal mis-coding errors, which replace a correct amino acid with an incorrect one but maintain the protein synthesis reading frame, +1-shifts move the ribosome to the next nucleotide in the 5′ to 3′ direction of the mRNA, resulting in loss of the reading frame, premature termination of protein synthesis, and ultimately cell death. The ability of m^1^G37-tRNA to suppress ribosomal +1-shifts is an important finding that has activated in-depth mechanistic studies with high significance. mRNA sequences such as the pyrimidine-ending CCC and CCU codons for proline (Pro), followed by another pyrimidine, resulting in CC[C/U]-[C/U] sequence motifs, are particularly prone to +1-shifts (Yourno and Tanemura, 1970) and are often found within the first 15 codons of protein-coding genes (Gamper et al., 2015a), where ribosomal translation is highly sensitive to attenuation (Chen and Inouye, 1990). Additionally, some of the +1-shift prone sequences are directly adjacent to the start codon (Gamper et al., 2015a), at the second codon position of the reading frame, where ribosomal translation is in the unique stage of transitioning from the initiation to the elongation phase.

The maintenance of protein synthesis reading frame in normal cellular conditions is achieved with unexpectedly high fidelity. Despite the dynamics of each ribosome that successively move tRNA-mRNA complexes from the A- to P- to E-site, and despite the rapid rate of protein synthesis at 10–20 amino acids per second, +1-shifts typically occur in less than one per 30,000 amino acids (Jorgensen and Kurland, 1990), at least 10-fold lower relative to other types of translational errors. In a genetic reporter system developed in *E. coli*, m^1^G37-tRNA was found to have the strongest suppression power when the shift-prone CCC-C sequence was placed next to the start codon (Gamper et al., 2015a). Lack of m^1^G37 in tRNA^Pro^ at this second-codon position increased +1-shifts by almost 10-fold in the reporter system, higher than increases of +1-shifts at any other position throughout the reporter gene. An *in vitro* assay was then developed using reconstituted *E. coli* ribosomes, with the goal to assess the rate of +1-shifts relative to the rate of peptide bond formation (Gamper et al., 2015a). Both fast and slow mechanisms were uncovered (Gamper et al., 2015a). The fast mechanism occurs on a timescale comparable to that of peptide bond formation. It takes place when tRNA^Pro^, carrying the first peptide bond made at the A-site, is moving into the P-site by a process known as translocation (Figure 1B). Thus, due to the ability of a +1-shift to compete with peptide bond formation on a similar timescale, should this process happen, it would compromise the accuracy of the reading frame. The slow mechanism occurs on a timescale 100-fold slower relative to peptide bond formation (Gamper et al., 2015a). It takes place when tRNA^Pro^, carrying the first peptide bond, is sitting at the P-site next to an empty A-site (Figure 1C), which is usually the case during nutrient starvation that depletes charged aminoacyl-tRNAs. The model that has emerged from these mechanistic studies is that the ribosome is most prone to +1-shifts after it completes the first peptide bond formation and is moving tRNA^Pro^ from the A- to the P-site. This is also the transitioning point of the ribosome from the initiation phase (where the first peptide bond is made) to the elongation phase (where the second peptide bond will be made when the third codon enters the ribosome A-site). The finding that m^1^G37 has the strongest suppression of +1-shifts at the second codon position indicates its pivotal role in maintaining the reading frame during the transition point of the ribosome. Should m^1^G37 be eliminated leading to ribosomal +1-shifts at the transition point, this would generate a prematurely terminated peptide in a non-productive and energetically costly translational error.

## Codon-Specific Translation by m^1^G37-tRna

Of the four CCN codons for Pro, CC[C/U] codons are most dependent on the presence of m^1^G37 (Jorgensen and Kurland, 1990) and the translation of these codons is noticeably slowed down upon deficiency of the methylation (Bjork and Nilsson, 2003). The codon-anticodon base-pairing interaction for CC[C/U] codons was thought to involve quadruplet base-pairing as implicated in previous studies (Hohsaka et al., 2001; Taki et al., 2002). However, there is no structural evidence of quadruplet pairing at the ribosomal A-site (Maehigashi et al., 2014) and the isolation of suppressor mutations of +1-shifts that are not in tRNA sequence suggests that quadruplet pairing is unlikely (Qian et al., 1998; Farabaugh, 2000). More recent work favors a model of tRNA slippage by a triplet codon-anticodon pairing interaction (Qian et al., 1998), which is supported by a detailed kinetic analysis (Gamper et al., 2015a). In the triplet slippage model, the +1-shift-prone sequences CC[C/U]-[C/U] are each read by two isoacceptors of tRNA^Pro^: the GGG isoacceptor exploits wobble pairing without additional modifications, whereas the UGG isoacceptor requires the 5-carboxy-methoxy (cmo^5^) modification to U34 at the wobble position (Nasvall et al., 2004). In all cases, the codon-anticodon pairing interaction in the unshifted 0-frame and in the shifted +1-frame is similar (Figures 1D,E), indicating minimum energetic penalty for the tRNA to shift. The role of m^1^G37 in suppressing the shift is to re-organize the structure of the anticodon loop to stabilize the pairing in the 0-frame (Maehigashi et al., 2014). A differential effect of m^1^G37 in suppressing the shift between the GGG and UGG isoacceptors is that the methylation by itself is insufficient in the GGG isoacceptor and requires the assistance of the elongation factor EF-P (Gamper et al., 2015a), whereas it is dominant in the UGG isoacceptor (Gamper et al., 2015a,b). In contrast, the dependence on m^1^G37 for reading-frame accuracy is much reduced with the purine-ending CC[G/A] codons for Pro, which are read by two isoacceptors of tRNA^Pro^: the CGG isoacceptor for the CCG codon and the UGG isoacceptor for both. Notably, the codon-anticodon pairing interaction in the +1-frame is highly unfavorable relative to the 0-frame, indicating that CC[G/A] codons are intrinsically more stable with pairing of the anticodon and are less likely to shift and less sensitive to m^1^G37 deficiency. This comparison illustrates the notion that synonymous codons have different requirements for pairing with the anticodon, and that such differences are the underlying basis of codon-specific translation.

The discovery that m^1^G37 has differential control of the translation of Pro codons is intriguing. Pro is a unique amino acid that contains an α-imino, rather than an α-amino group. It is the slowest substrate relative to all other amino acids for peptide bond formation, both as the acceptor or the donor of making a peptide bond by the ribosome (Pavlov et al., 2009). The aminoacyl group of Pro when charged to tRNA^Pro^ is also the least stable compared to others and it has the lowest affinity to the elongation factor EF-Tu (Peacock et al., 2014). Additionally, Pro is the only amino acid that can enable peptide backbones to make turns and change direction, so its position and distribution in a protein sequence directly impact on folding of the protein, particularly if trans-membrane domains are involved (Yohannan et al., 2004). Also, human disease-associated mutations are frequently found at Pro positions (Partridge et al., 2004). These considerations suggest that m^1^G37-tRNA has the ability to regulate a diverse process of cellular activities via differential translation of Pro codons at specific positions during gene expression.

Besides the universal association of m^1^G37 with all isoacceptors of tRNA^Pro^, the CCG isoacceptor of tRNA^Arg^ for reading the Arg CGG codon, and the GAG isoacceptor of tRNA^Leu^ for reading the Leu CU[C/U] codons, also contain m^1^G37. The mechanism for why these isoacceptors carry m^1^G37 has not been investigated. It does not appear to involve suppression of +1-shifts, based on analysis of the codon-anticodon pairing interaction, but may involve release of ribosomes from stalling at the specific codons. Notably, the m^1^G37-containing CCG tRNA^Arg^ is only one of the several isoacceptors of the Arg family, whereas other members of the family do not need m^1^G37. Similarly, while m^1^G37 is present in the GAG, CAG, and UAG isoacceptors of the Leu family, it is absent from the UAA isoacceptor. This provides additional evidence for codon-specific translation that is dependent on the presence of post-transcriptional modifications in the anticodon for pairing with a codon.

## Codon-Specific Translation in Mg^2+^ Homeostasis

**FIGURE 2:**
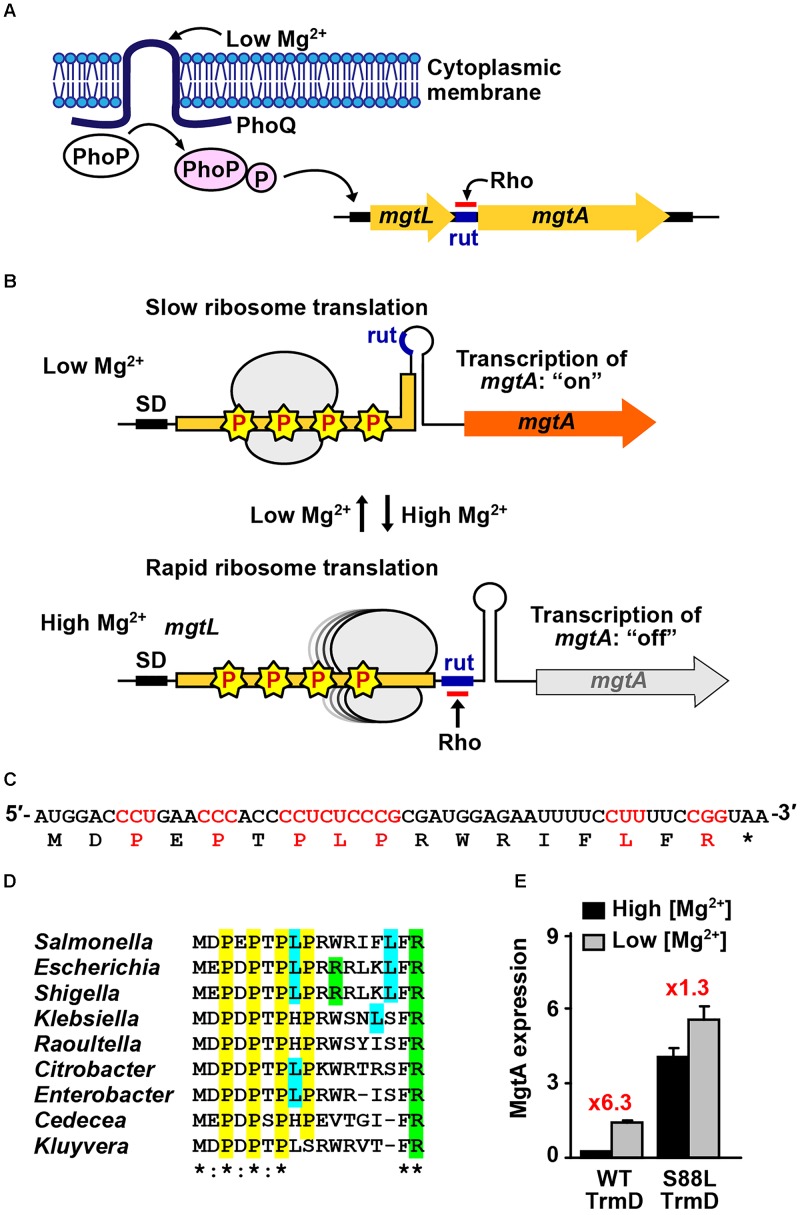
Codon-specific translation in Mg^2+^ homeostasis. **(A)** Mg^2+^ homeostasis in *Salmonella* is maintained by the membrane-bound two-component system PhoPQ sensing of the external low Mg^2+^, which activates transcription of the major transporter gene *mgtA*. Transcription of *mgtA* is determined by ribosomal translation of the 5′-leader ORF, which contains several m^1^G37-dependent Pro codons. **(B)** Low levels of Mg^2+^ slow down ribosomal translation due to stalling at m^1^G37-dependent codons, resulting in a structure of the 5′-leader ORF that places rut in a stem-loop region inaccessible to Rho, thus allowing transcription through *mgtA*, whereas high levels of Mg^2+^ enable rapid ribosomal translation of the 5′-leader ORF, which exposes the rut sequence ahead of the *mgtA* structure gene and attenuates transcription. **(C)** The codon sequence of the 5′-leader ORF is shown, where m^1^G37-dependent codons are highlighted. Asterisk “^∗^” indicates a termination codon. **(D)** The m^1^G37-dependent codons in the 5′-leader ORF are highly conserved across different species of Gram-negative bacteria. Asterisk “^∗^” indicates complete conservation among all the sequences, whereas a colon “:” indicates conservation between those with strongly similar properties. **(E)** *Salmonella* cells expressing the native *trmD* show a robust response of activation of transcription of *mgtA* from high to low Mg^2+^ (6.3-fold), whereas cells expressing a mutant *trmD* show a diminished response (1.3-fold), consistent with codon-specific translation at m^1^G37-dependent codons in the 5′-leader ORF. Data are obtained from published work (Gall et al., 2016).

An example of codon-specific translation mediated by m^1^G37 is the maintenance of Mg^2+^ homeostasis in Gram-negative bacteria. Mg^2+^ is the most abundant divalent cation in all living cells and is maintained at mM concentrations. For *Salmonella enterica* serovar Typhimurium (hereafter *Salmonella*), the etiologic agent of human gastroenteritis, the infection of the human gut into the metal-scarce macrophage compartment, requires Mg^2+^ transport into cells for survival and virulence of the pathogen (Papp-Wallace and Maguire, 2008a; Groisman et al., 2013). This transport is activated upon expression of the major Mg^2+^ transporter gene *mgtA* and is regulated at two levels: the transcriptional activation of the structural gene and the ribosomal translational control of the 5′-leader ORF ahead of the structural gene (Figure 2A). First, the initial transcription activation is by the membrane-bound two-component PhoPQ system, in which sensing of low external Mg^2+^ by PhoQ promotes phosphorylation of PhoP, which activates transcription of *mgtA* and many virulence genes (Papp-Wallace and Maguire, 2008a; Groisman et al., 2013). Second, the subsequent transcriptional regulation of *mgtA* is determined by the speed of ribosomal translation of the 5′-leader ORF of the *mgtA* mRNA. Rapid translation of this ORF exposes the Rho-utilization (rut) sequence, resulting in attenuation of transcription before the *mgtA* gene (Cromie et al., 2006). In contrast, slow or stalled translation of the ORF induces a structural change in the 5′-leader mRNA that places the rut sequence inaccessible to Rho-dependent termination, thus allowing transcription through *mgtA* (Park et al., 2010; Hollands et al., 2014; Figure 2B). This translation-dependent attenuation of transcription is common to regulation of expression of amino-acid biosynthesis genes (Merino and Yanofsky, 2005). In addition, *Salmonella* has a second inducible Mg^2+^ transporter gene *mgtB* expressed from the virulence operon *mgtCBR* under a similar control of transcriptional attenuation that is determined by the speed of ribosomal translation of its 5′-leader ORF (Papp-Wallace and Maguire, 2008a; Groisman et al., 2013). While *Salmonella* has a third and constitutively expressed Mg^2+^ transporter gene *corA* (Papp-Wallace and Maguire, 2008b), it is the inducible expression of *mgtA* and *mgtB* that maintains Mg^2+^ at virtually constant levels. While the external Mg^2+^ level can change by 5 orders of magnitude, the internal level varies by less than fivefold (Papp-Wallace and Maguire, 2008b). Without this Mg^2+^ homeostasis, *Salmonella* cannot survive in host cells. Thus, the translation-dependent attenuation of transcription of *mgtA* is the major determinant of Mg^2+^ homeostasis for *Salmonella*.

Analysis of the 5′-leader ORF of *mgtA* shows a strong bias for codons that are dependent on m^1^G37-tRNA for translation (Park et al., 2010). These include Pro codons CC[C/U] at positions 3, 5, and 7, Leu codons CU[C/U] at positions 8 and 15, and Arg codon CGG at position 17 (Figure 2C). These m^1^G37-dependent codons are highly conserved across diverse Gram-negative bacteria (Figure 2D), indicating significance under evolutionary pressure. The codon conservation raises the possibility of codon-specific translation, where the speed of ribosomal translation of these codons is controlled by the presence of m^1^G37. A strong support for this possibility is that TrmD, the bacterial methyl transferase that synthesizes m^1^G37, is strictly dependent on Mg^2+^ for catalytic activity (Sakaguchi et al., 2014). While the requirement for Mg^2+^ for several tRNA methyl transferases is known (Hurwitz et al., 1964; Kumagai et al., 1982), the requirement in TrmD is strictly at the transition state of the catalytic mechanism (Sakaguchi et al., 2014), which is an unexpected finding that links the synthesis of m^1^G37 to cellular concentrations of the metal ion.

In a genetic reporter system, where *mgtA* was fused to *lacZ*, the transcription of *mgtA* was monitored in *Salmonella* cells grown in media containing high or low Mg^2+^ (1.6 vs. 0.016 mM) (Gall et al., 2016). It was found that cells expressing the native *trmD* showed more than a sixfold activation of transcription upon switching from high to low Mg^2+^ media, whereas cells expressing a mutant *trmD* showed less than a twofold activation (Figure 2E). The mutant *trmD* harbors a mutation (S88L) near the AdoMet binding site (Masuda et al., 2013), which prevents the enzyme from binding to the methyl donor and from performing the Mg^2+^-dependent methyl transfer. The reported observation supports a model of codon-specific translation in the 5′-leader ORF. For cells expressing the native *trmD*, the level of Mg^2+^ modulates the level of TrmD-dependent m^1^G37-tRNA synthesis, which in turn modulates the speed of ribosomal translation of m^1^G37-dependent codons in the 5′-leader ORF. At high Mg^2+^, TrmD is active and the abundantly synthesized m^1^G37-tRNA facilitates ribosomal translation through the 5′-leader ORF, thus attenuating the transcription of *mgtA*. At low Mg^2+^, by contrast, TrmD is inactive, m^1^G37-tRNA synthesis is reduced, and ribosomal translation of m^1^G37-dependent codons is stalled, thus activating transcription through the *mgtA* gene and producing a robust response. This response to changes of Mg^2+^ concentrations is reduced in cells expressing the mutant *trmD*, consistent with the observed lower level of activation of *mgtA* transcription.

## Perspective

Codon bias has the ability to regulate protein expression by controlling the efficiency or accuracy of protein synthesis. Codon bias can be executed by post-transcriptional modifications of the tRNA anticodons that determine the quality of pairing interaction with the cognate codons at local positions. While it is well documented that altering the codon usage synonymously can alter the expression levels of the manipulated genes (Kudla et al., 2009; Navon and Pilpel, 2011; Goodman et al., 2013; Zhou et al., 2013), much less is known how the alteration is correlated with post-transcriptional modifications of the tRNA anticodons in response to changes of the codon usage. While we present one example in the regulation of bacterial Mg^2+^ homeostasis by the m^1^G37 modification of tRNA (Gall et al., 2016), there are increasing studies demonstrating the ability of post-transcriptional modifications in the anticodon region to alter protein expression. Examples include cmo^5^U34 (Chionh et al., 2016), mcm^5^U34 (Begley et al., 2007), and Q34 (Tuorto et al., 2018) to the wobble position, and t^6^A37 on the 3′-side of the anticodon (Lin H. et al., 2018), all of which are induced in response to stress, indicating the ability to reprogram the proteome during cellular adaptation to stress. More broadly, even modifications that are outside of the anticodon but are important for tRNA stability can regulate protein expression by altering the abundance of a specific tRNA, thus impacting on the progress of disease. Examples include m^1^A58 in the T loop (Richter et al., 2018) and m^7^G46 in the V loop (Lin S. et al., 2018). The diversity of post-transcriptional modifications of tRNA is a key feature of the importance of tRNA biology. We are only at the tip of the iceberg at the forefront of exciting new discoveries.

## Author Contributions

Y-MH prepared the manuscript. IM prepared the figures. HG contributed to discussion. All authors made a substantial intellectual contribution to the work and approved its presentation.

## Conflict of Interest Statement

The authors declare that the research was conducted in the absence of any commercial or financial relationships that could be construed as a potential conflict of interest.
